# Supplementary material for: Genomic Diversity of SARS-CoV-2 During Early Introduction into the United States National Capital Region
Source: medRxiv. 2020 Aug 23:2020.08.13.20174136. Originally published 2020 Aug 15. Preprint. [Version 2] doi: 10.1101/2020.08.13.20174136 (PMC7430609; doi:10.1101/2020.08.13.20174136)
Supplement: 1 [file NIHPP2020.08.13.20174136-supplement-1.pdf]

medRxiv preprint doi: https://doi.org/10.1101/2020.08.13.20174136.this version posted August 23, 2020. The copyright holder for this preprint (which was not certified by peer review) is the author/funder, who has granted medRxiv a license to display the preprint in perpetuity.
It is made available under a CC-BY 4.0 International license .

## Supplemental Figures and Tables

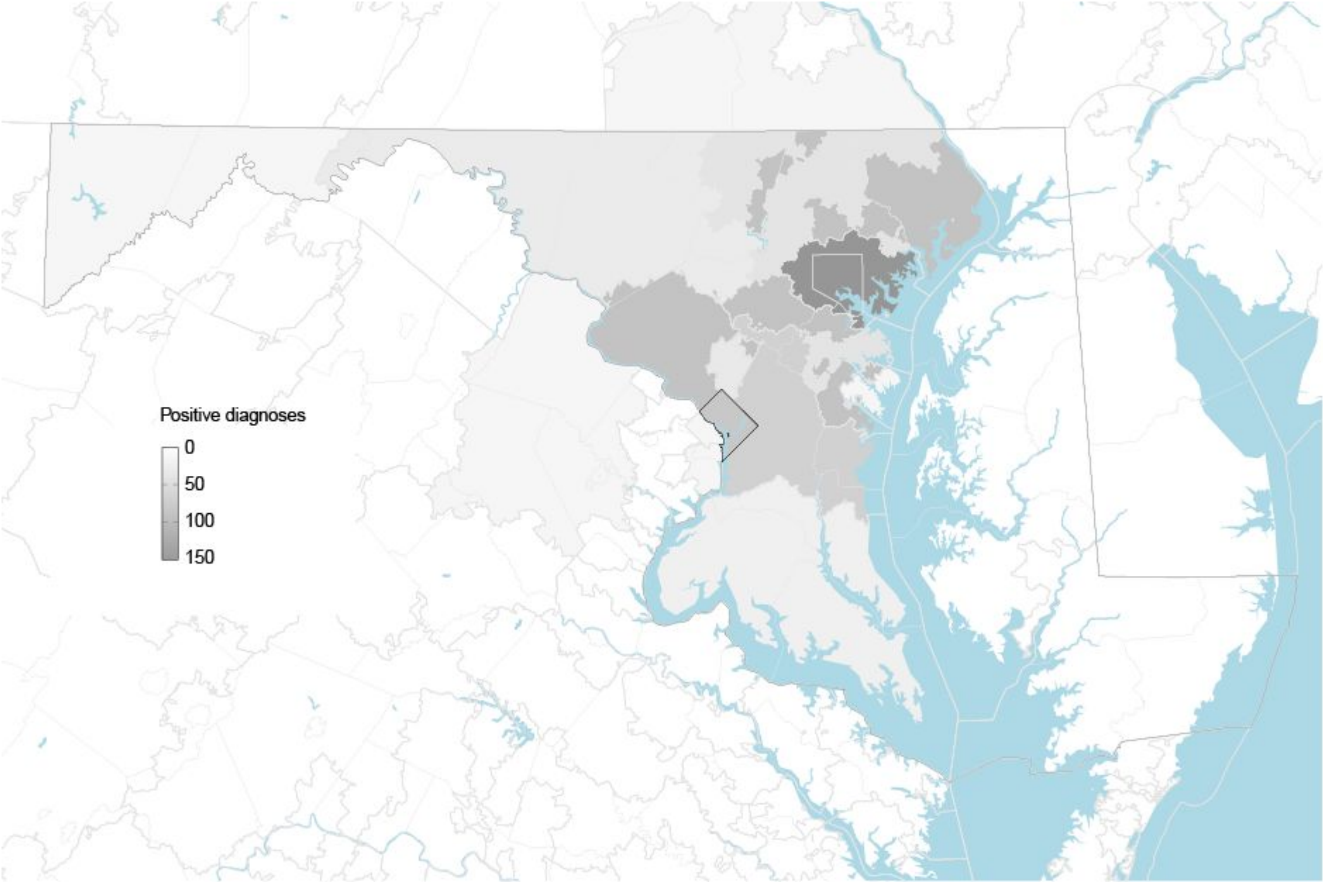


**Supplemental Figure 1. Positive diagnoses by first three digits of zip code.** Outlined regions correspond to areas sharing the first three digits of their zip code (Washington DC outlined in black, all others grey). Each region is shaded by the number of patients with positive COVID-19 diagnoses in March 2020 (from the Johns Hopkins Health System) reporting home residence in that region. Regions with 1–5 positive diagnoses are shown as 5.

medRxiv preprint doi: https://doi.org/10.1101/2020.08.13.20174136.this version posted August 23, 2020. The copyright holder for this preprint (which was not certified by peer review) is the author/funder, who has granted medRxiv a license to display the preprint in perpetuity.
It is made available under a CC-BY 4.0 International license .

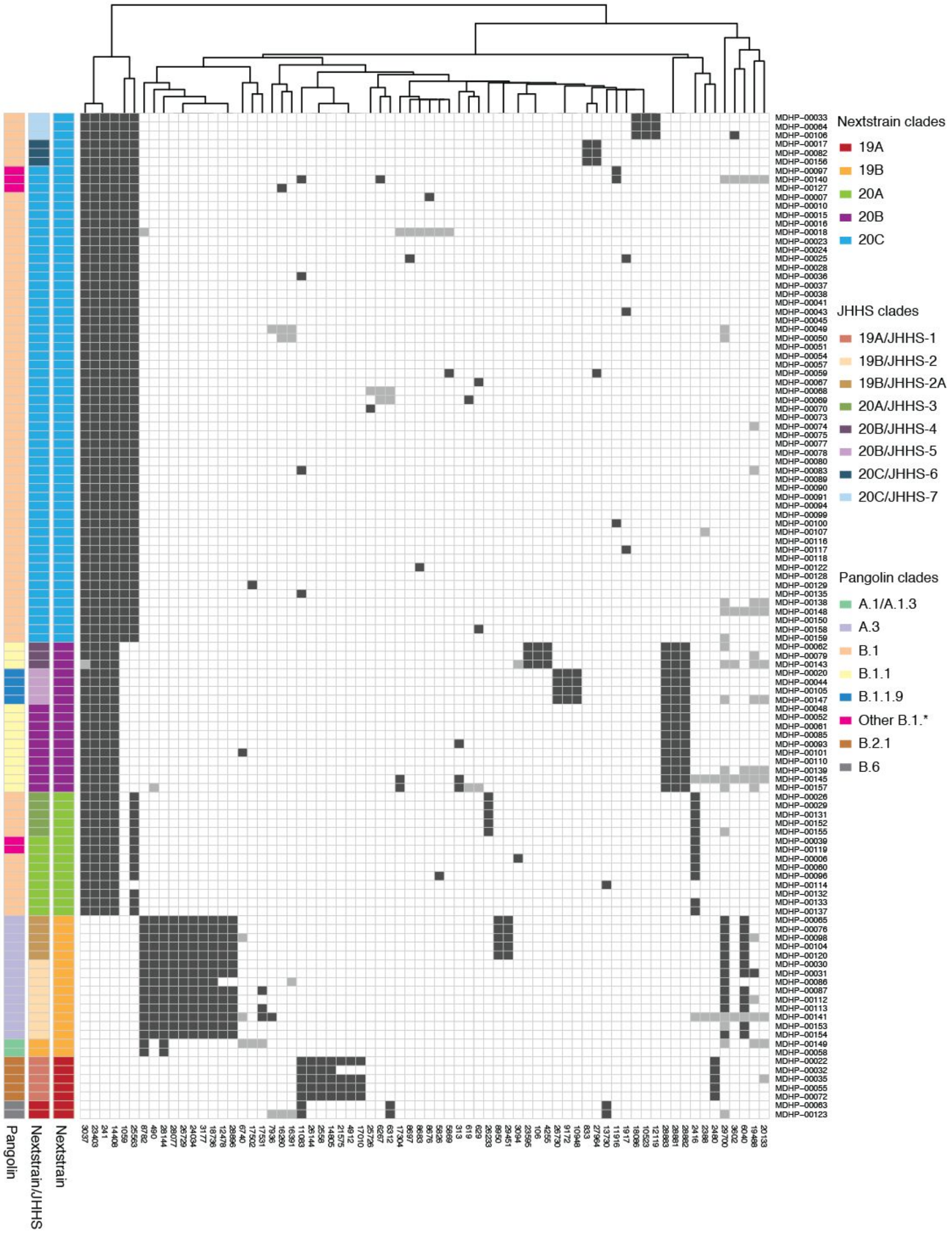


**Supplemental Figure 2. Single nucleotide polymorphisms present in three or more JHHS sequences.** Rows = samples sequenced as part of this study (114), sorted by Nextstrain clade. Columns = positions with consensus variants in three or more sequences. Black cells = variant present; grey cells = ambiguity present. Columns are clustered using complete linkage hierarchical clustering. JHHS subclades are additional subclades of Nextstrain clades in which two or more variants are shared between three or more JHHS sequences. Some JHHS-specific clades are captured by Pangolin nomenclature, some are not.

medRxiv preprint doi: https://doi.org/10.1101/2020.08.13.20174136.this version posted August 23, 2020. The copyright holder for this preprint
(which was not certified by peer review) is the author/funder, who has granted medRxiv a license to display the preprint in perpetuity.
It is made available under a CC-BY 4.0 International license .

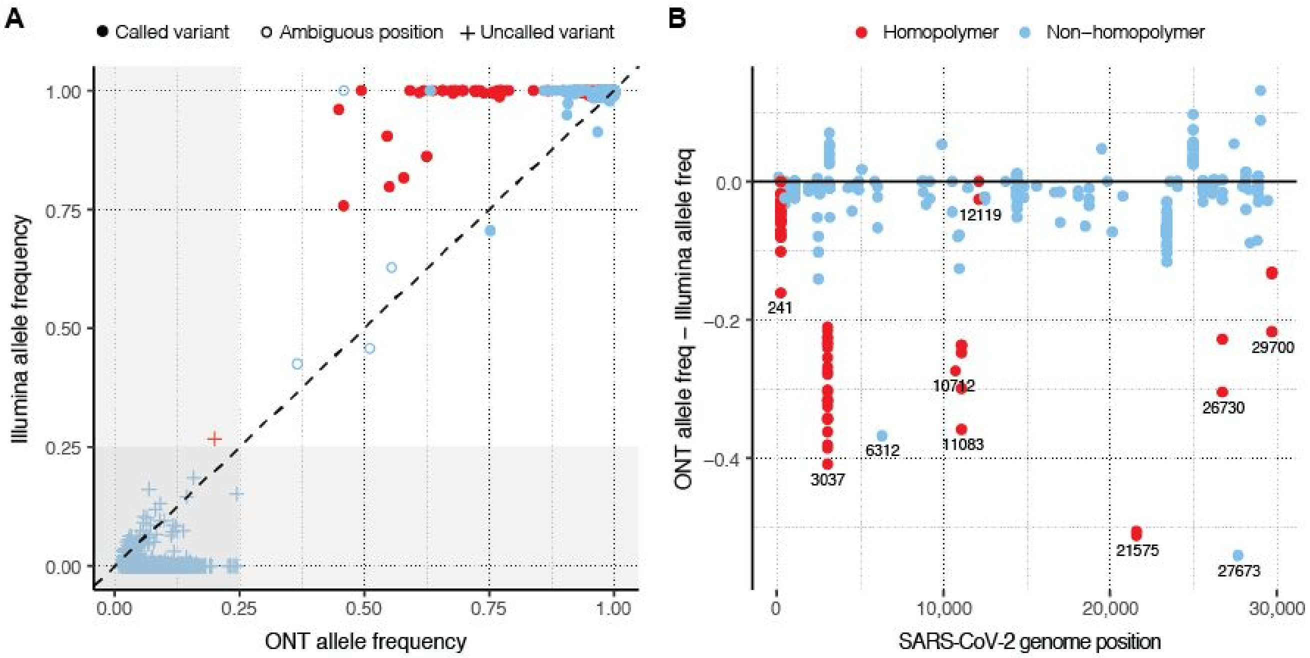


**Supplemental Figure 3. Illumina and Oxford Nanopore allele frequency comparison.** (**A**) Variant frequencies from tiled amplicons sequenced on the Oxford Nanopore (ONT) and Illumina platforms. All variants at >0.02 allele frequency are shown. Uncalled variants are those not present in the consensus sequences from these samples. Grey shaded regions represent frequency threshold (25%) used to automatically reject candidate variants. Almost all discrepancies in frequency occur within homopolymer regions (red symbols). (**B**) Difference between allele frequencies across the two sequencing platforms. Position 3,037 (within a T-homopolymer) accounts for most large discrepancies in frequency.

medRxiv preprint doi: https://doi.org/10.1101/2020.08.13.20174136.this version posted August 23, 2020. The copyright holder for this preprint (which was not certified by peer review) is the author/funder, who has granted medRxiv a license to display the preprint in perpetuity.
It is made available under a CC-BY 4.0 International license .

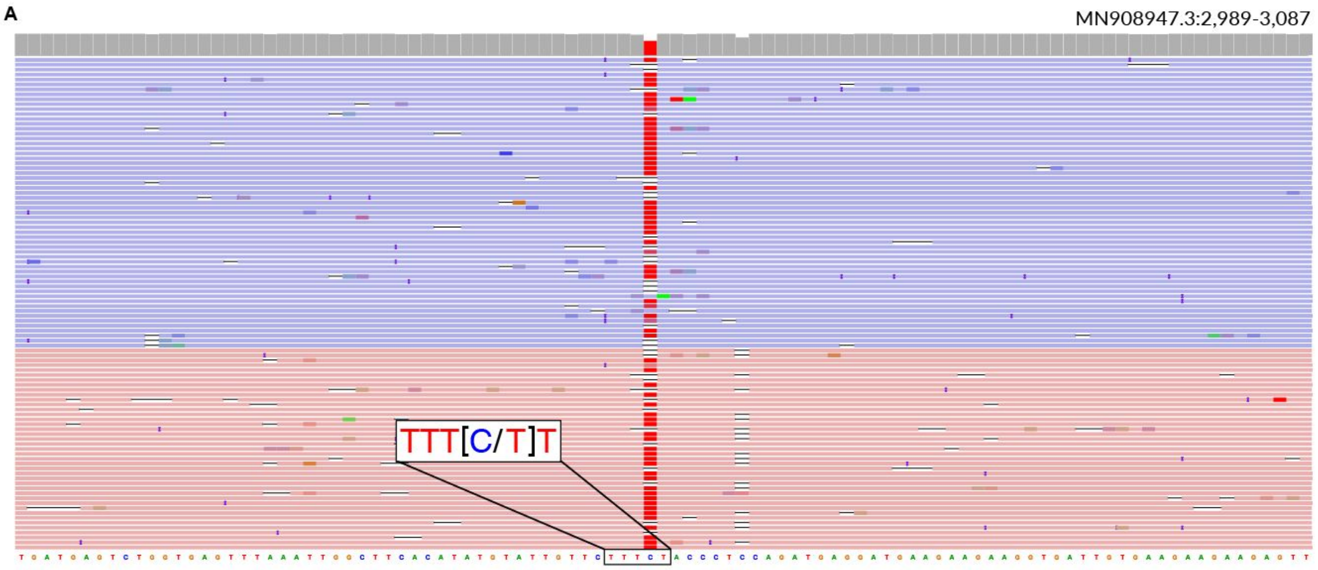


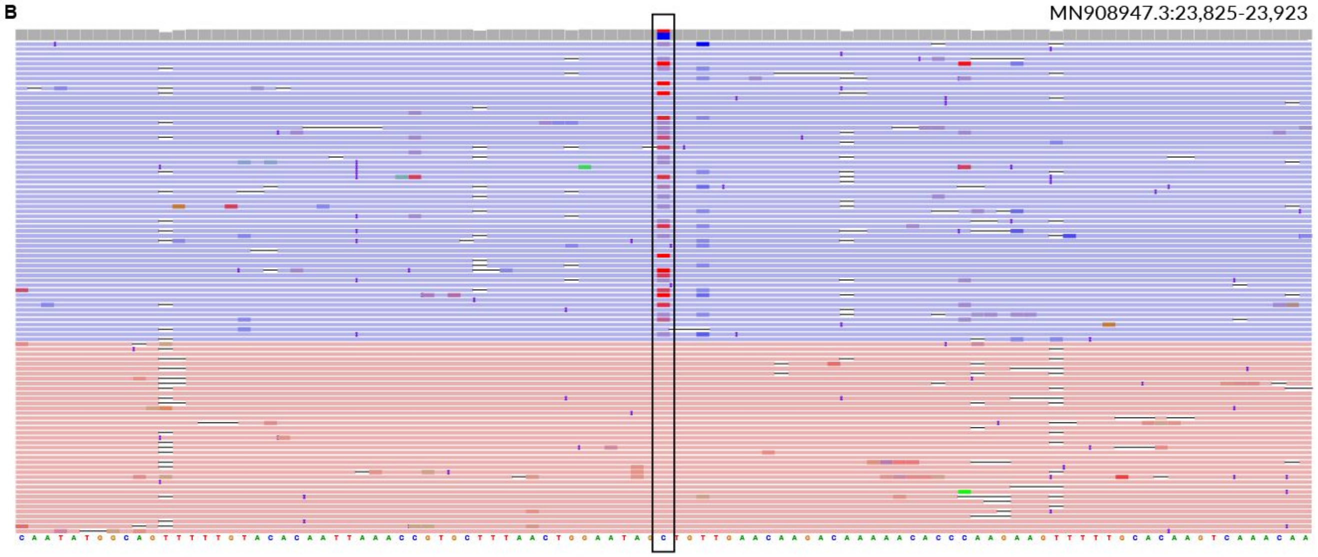


**Supplemental Figure 4. Common Oxford Nanopore sequencing issues.** (**A**) Example of a T-homopolymer region in the SARS-CoV-2 genome (sample MDHP-00028). A large number of reads have deletions, resulting in a mixed alternate allele frequency. (**B**) Example of strand bias in Oxford Nanopore sequencing reads (sample MDHP-00028). A mutation only occurs in minus strand reads.

medRxiv preprint doi: https://doi.org/10.1101/2020.08.13.20174136.this version posted August 23, 2020. The copyright holder for this preprint (which was not certified by peer review) is the author/funder, who has granted medRxiv a license to display the preprint in perpetuity.
It is made available under a CC-BY 4.0 International license .

Genetic distance ($d$) ($\times 10^{-4}$)

| | JHHS-DC (n=31) | DC other (n=6) | DC (n=37) | JHHS-MD (n=83) | MD other (n=8) | MD (n=91) | VA (n=50) | LA (n=34) | ID (n=32) | NY (n=35) | CA (n=53) | WA (n=61) | Global 1K (n=886) | Global 3K (n=2593) |
|---|---|---|---|---|---|---|---|---|---|---|---|---|---|---|
| **JHHS-DC** | **2.25** | | | | | | | | | | | | | |
| **DC other** | 2.10 | **2.00** | | | | | | | | | | | | |
| **DC** | 2.17 | 2.03 | **2.20** | | | | | | | | | | | |
| **JHHS-MD** | 3.13 | 2.88 | 3.09 | **3.73** | | | | | | | | | | |
| **MD other** | 2.23 | 2.07 | 2.21 | 3.16 | **1.83** | | | | | | | | | |
| **MD** | 3.05 | 2.81 | 3.01 | 3.64 | 3.03 | **3.62** | | | | | | | | |
| **VA** | 2.77 | 2.47 | 2.72 | 3.47 | 2.73 | 3.41 | **2.95** | | | | | | | |
| **LA** | 2.19 | 2.09 | 2.18 | 3.17 | 2.19 | 3.08 | 2.89 | **1.32** | | | | | | |
| **ID** | 2.32 | 2.15 | 2.29 | 3.26 | 2.29 | 3.17 | 2.93 | 1.68 | **1.52** | | | | | |
| **NY** | 2.47 | 2.26 | 2.43 | 3.34 | 2.42 | 3.26 | 3.02 | 2.39 | 2.49 | **2.51** | | | | |
| **CA** | 3.06 | 2.61 | 2.99 | 3.62 | 3.21 | 3.59 | 3.13 | 3.38 | 3.36 | 3.31 | **2.64** | | | |
| **WA** | 4.20 | 3.39 | 4.07 | 4.53 | 4.43 | 4.52 | 3.84 | 4.62 | 4.55 | 4.41 | 3.11 | **2.57** | | |
| **Global 1K** | 3.09 | 2.79 | 3.04 | 3.71 | 3.24 | 3.67 | 3.31 | 3.43 | 3.41 | 3.37 | 2.98 | 3.74 | **3.16** | |
| **Global 3K** | 3.00 | 2.73 | 2.96 | 3.65 | 3.13 | 3.60 | 3.27 | 3.31 | 3.30 | 3.28 | 3.04 | 3.86 | 3.17 | **3.16** |

**Supplemental Figure 5. Evolutionary divergence in geographic regions.** Pairwise matrix containing average pairwise genetic distances within (bolded in the diagonal) and between sequences from various geographic regions including: the District of Columbia (JHHS-DC + DC other), Maryland (JHHS-MD + MD other), Virginia (VA), Louisiana (LA), Idaho (ID), New York (NY), California (CA), Washington (WA), and two representative global subsamples (Global 1K and 3K). Green-to-red color scale highlights the most (red) and least (green) divergent within or between average distances.

**Supplemental Table 1. Aggregate JHHS diagnostic tests by date.**

**Supplemental Table 2. Aggregate sample metadata.**

**Supplemental Table 3. Sequencing metrics.**

**Supplemental Table 4. Single nucleotide polymorphisms present in JHHS sequences.**

**Supplemental Table 5. Accession numbers of samples used in phylogenetic analysis.**

**Supplemental Table 6. Accession numbers of samples used in genetic distance analysis.**
